# Children's liking and wanting of snack products: Influence of shape and flavour

**DOI:** 10.1186/1479-5868-6-38

**Published:** 2009-07-02

**Authors:** Djin G Liem, Liesbeth H Zandstra

**Affiliations:** 1 Deakin University, School of Exercise and Nutrition Sciences. Faculty of Health, Medicine, Nursing and Behavioural Sciences, 221 Burwood Highway, Burwood VIC 3125, Australia; 2 Unilever R&D Vlaardingen, Sensation Perception & Behaviour, Olivier van Noortlaan 120, PO Box 114, 3130 AC Vlaardingen, the Netherlands

## Abstract

**Background:**

Children's food choices are guided by their preferences. However, these preferences may change due to repeated exposure.

**Methods:**

This study investigated children's (n = 242, 7–12 yrs-old) liking and wanting for snacks over 3 weeks of daily consumption. The snacks differed in size (small vs large) or flavour (sweet vs sweet-sour). Two conditions were designed: 1) a monotonous group in which children continuously consumed the same snack across the 3 weeks, and 2) a free choice group in which children were allowed to freely choose amongst 3 different flavours of the snack each day during 3 weeks.

**Results:**

Shape influenced long-term liking, i.e. small shaped snacks remained stable in liking over repeated consumption, whereas large shaped snacks with the same flavour decreased in liking. Mean wanting ratings for all snack products decreased over 3 weeks daily consumption. Flavour did not significantly influence liking and wanting over time. The ability to freely choose amongst different flavours tended to *decrease *children's liking (p < 0.1) and wanting (p < 0.001) for these products. Changes in liking rather than initial liking was the best predictor of snack choice during the intervention.

**Conclusion:**

Wanting rather than liking was most affected by repeated daily consumption of snack foods over three weeks. In order to increase the likelihood that children will repeatedly eat a food product, smaller sized healthy snacks are preferred to larger sized snacks. Future research should focus on stabilizing wanting over repeated consumption.

## Background

Children's liking of the taste of a product has been identified as the most important determinant of children's food choice [1-3]. Several tests have been developed to measure children's liking (see [4] for review). Most of these tests use a one-off tasting to predict which products children like best. However, the taste children like (i.e. liking for sweet and dislike for bitter taste) changes during the life span [5-7] and across weeks, due to for example repeated exposure.

It has been argued that repeated exposure to a particular food can lead to an increase in liking of this food. It needs, however, to be noted that most of these studies were performed with foods which were quite different from each other (e.g. different vegetables or fruits) [8] or were novel to the child [9]. This makes it difficult to determine which specific product properties (e.g. flavour profile, appearance, size) are important for a change in liking after repeated exposure.

It could be that liking for some products increases after repeated exposure, whereas liking for other products remain stable or decreases after repeated exposure. Liem et al [10] found that repeated exposure (i.e. 8 days, once a day) to a sweet drink increased children's liking for this drink. In contrast, repeated exposure to a sour drink, which was at the start similarly liked as the sweet drink, remained stable in liking. It remains to be investigated whether sweet-sour balance also plays a role in changes in liking for solid foods after a daily exposure during several weeks.

In adults changes in liking after repeated exposure has been investigated extensively. In these studies repeated exposure generally did not result in an increase in liking but rather a decrease in liking. This has been referred to as *boredom *or monotony, which can be defined as the lowered acceptance of a food as a function of the number of times a food is consumed (Sigel & Pelgrim, 1958 in [11]). Boredom can be caused by either neurophysiological responses, i.e. a decrease in actual liking caused by satiation with specific attributes of the consumed food, and/or cognitive response, i.e. a decrease wanting to eat the food [12,13]. These two causes have previously been set out as liking vs wanting. *Liking *can be defined as the pleasure derived from oro-sensory stimulation of food. *Wanting *can be defined as incentive salience, the motivation to engage in eating [14]. Extensive animals research by Berridge suggests that liking and wanting have separate neural substrates (i.e. dopamine vs opioid) and can act independently. This has been replicated in humans by using specific dopamine and opioid antagonists (see [15] for review). It has been suggested that liking and wanting play an important interdependent role in food choice and consumption in adults [14,16].

Studies which focussed on children's liking *and *wanting as separate pathways for food choice are scarce. Previous studies either measured liking and wanting as one concept [17,18], only measure one of the two pathways [19,20], or did not investigate which product properties are associated with a decrease in liking and wanting [21]. In order to investigate changes in liking and wanting for foods children have repeatedly been exposed to, we may learn from research conducted with adults.

It has been argued that the size of the food eaten plays an important role in the decrease of liking and wanting after repeated exposure. A recent study of Weijzen *et al*. suggested that after repeated consumption of small snack foods a statistically significant decrease in wanting but not in liking was observed. After a repeated consumption of large snack foods a statistically significant decrease in wanting *and *liking was observed [22]. They argued that the oral sensory stimulation that positively relates to the size of the food is related to liking and wanting. In this study, however, they investigated changes in liking and wanting within one meal consumption rather than over an extended period of time. It remains to be investigated whether size also influences liking and wanting after daily consumption of these foods for several weeks.

Furthermore, it has been suggested that a decrease in liking and wanting (measured as boredom) after repeated exposure can be minimized by giving adult [12,23] or children [21] a choice between different products. Hypothetically, in a choice situation participants have a larger feeling of control of what they eat, which decreases the perceived boredom [12].

The current study investigated three hypothesis related to children's change in liking and wanting. The first hypothesis concerned the influence of sweet-sour balance on children's liking and wanting. It was hypothesised that after daily consumption for three weeks, the liking and wanting of sweet snack foods would increase and the liking and wanting of sour snack foods would remain stable. This was tested by means of snack products which flavours were either Sweet or Sweet-Sour.

The second hypothesis concerned the influence of snack size on children's liking and wanting. It was hypothesised that Small sized snacks (e.g. nibbles) resulted in less decrease of liking but not wanting over daily consumption for three weeks than Large sized snack (e.g. bars). This was tested by means of snack products which differed in size.

The third hypothesis concerned the influence of choice on children's liking and wanting. It was hypothesised that children who could freely choose between snack products which differed in flavour and size would express a lower decrease in liking and wanting, over daily consumption for three weeks, than those who were not given a choice.

## Methods

### Participants

Children were recruited during door to door interviews in the Istanbul metro area in Turkey. Exclusion criteria were reported allergies for chocolate, polenta, sugar, dairy products, corn, corn oil, hazelnut or caramel. In addition, children were excluded from participation if they participated in any research concerning snack products in the past month. Initially 341 children started the study and 242 (n = 122, 7–9 yrs, n = 120, 10–12 yrs; 122 girls, 120 boys) completed the study. During the 3-week course of the study children dropped out of the study because of various reasons e.g. parents no longer gave permission or children did not want to participate any longer, failed to conduct the in-home liking test, or failed to give the products every day. The study was carried out according the ESOMAR ethical standards embodied in the ICC/ESOMAR Code of marketing and social research practise. Informed consent was obtain from the participants prior to participation

### Stimuli

The stimuli comprised of 5 snacks. Two had the same size (Small) but were different in flavour (Sweet vs Sweet-Sour flavour). Two had the same flavour (Chocolate Hazelnut) but were different in size (Small vs Large size). One snack was used as control-snack (Small size, caramel flavour). This product was only tasted during the baseline and end-measurement.

The Small sized snacks were on average 1.5 gram and 2.5 cm × 1.5 cm × 1.0 cm in size and presented in bags which contained 36 grams of snacks each (Unilever, Turkey). The Large size snack was on average 16.0 gram and 9.0 cm × 4.0 cm × 1.0 cm in size and presented in bags which contained 2 bars (Unilever, Israel). Both Small and Large snack foods comprised of a crunchy outer layer and a cream filling. The percentage cream relative to the weight of the snacks was kept constant. Per 100 gram the snacks contained 454 kcal- 63 carbohydrates, 9 gram protein and 16 gram fat. All stimuli were presented in non-labelled packs of aluminium foil which prevented light oxidation (see Table 1).

**Table 1:** Product characteristics (Flavour, maximum serving per day and abbreviation)

**Format**	**Flavour**	Maximum Serving per day*	**Abbreviation**
**Small**	Chocolate Hazelnut	1 bag = 32 gram	Small chocolate-hazelnut
**Large**	Chocolate Hazelnut	2 bars = 32 gram	Large chocolate-hazelnut
**Small**	Orange Bubble gum	1 bag = 32 gram	Small Sweet
**Small**	Orange Bubble gum with citric acid	1 bag = 32 gram	Small Sweet-Sour
**Small**	Caramel		Control

* each bag of nibbles contained about 20 nibbles

### Study design

#### Overview

**Figure 1:**
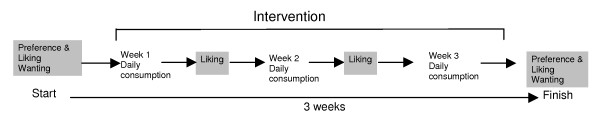
**Schematic overview of the procedure**. Measurements of liking (4 in total) and wanting (2 in total) are listed in grey blocks.

To test the hypotheses a between subject design was chosen. Children were asked to consume either Small chocolate-hazelnut, Large chocolate-hazelnut, Small sweet or Small sweet-sour snacks for a period of 3 consecutive weeks. Just before and at the end of the 3 weeks children's preference, liking and wanting for all snack foods were tested. After each week of exposure children's liking for snacks they consumed that week was tested (see Figure 1). Liking and wanting was individually measured in children's home by a trained interviewer. Parents were instructed to offer the snacks at the same time every day to minimize variation due to the time of day.

#### Group composition

Because in a real life situation children would choose to eat products they like, children were grouped based on their initial liking for the products, which resulted in 6 groups. Children who preferred the Small snack with Chocolate Hazelnut flavour either as their most or second most favourite were placed in the group that received this snack daily for three weeks (Group Small chocolate-hazelnut, n = 41). In a similar way a Large chocolate-hazelnut group (n = 41), a Small Sweet group (n = 40), and a Small Sweet-Sour group (n = 40) were composed. A fifth group, whose children were randomly chosen from the previous groups (before the intervention started), was given a free choice of three different Small snacks (Small chocolate-hazelnut, Small Sweet, Small Sweet-Sour) (hereafter referred to as CHOICE group, n = 40). Every day children in the CHOICE group were presented with three bags of small snacks of which they could choose one to consume, after sampling one snack from each pack. The 6^th ^group did not receive any snack foods other than at the beginning and the end of the intervention (hereafter referred to as CONTROL, n = 40).

### Sensory measures

#### Preference and liking

Preference was measured by means of a rank-order method. Children were presented with all the different snacks. The interviewer asked the child to taste all the snacks and point to the one he or she liked best. This snack was removed from the table after which the procedure was repeated with the remaining snacks until all were place in a rank-order from most to least liked [24]. The least liked snack was assigned 1 point, the most liked was assigned 5 points. All other snacks where given points between 1 and 5 according their position in the preference rank-order.

Subsequently, the researcher showed the child pictures of 5 different drawings of faces representing 1) extremely liked, 2) liked, 3) maybe liked, maybe disliked, 4) disliked, 5) extremely disliked. The researcher explained the meaning of the 5 faces by saying: *"This is the face that you make when you do not like something at all. This is a face you make when you do not like something. This is the face you make when you do not like it but also not dislike it. This is a face you make when you just like something. This is a face you make when you like something very much"*. Next, the child was asked to score the most preferred product on the 5-point facial scale. This procedure continued until all the stimuli were scored on liking. All children understood the procedure as suggested by the consistency between the ranking and scoring part. This procedure has been used and validated across different cultures such as France [25], UK [26], US [27].

#### Wanting

In order to obtain information about whether children's wanting for the product changed after three weeks of exposure, they were asked to taste the products and to rate them on how much they wanted to eat of it right now (i.e. really do not want to eat this-1 points; don't want to eat this-2 points; I do not know- 3 points; I want to eat it- 4 points; I really want to eat it- points). Previous research used similar explicit measurements of wanting [19,20].

#### Amount eaten

Children were free to consume any amount they wanted with a maximum of one bag of Small snacks, or two bars of the Large snack. Although snacks were offered every day children could decide not to consume the snacks at all. When children did not eat the whole bag or the two bars they were provided, parents were asked to save it. Each week the researchers collected and measured (grams) the left-overs. Due to logistic reasons the amounts consumed were measured on a group level rather than individual level.

### Data analyses

In order to determine significant differences between different products in initial liking and in initial wanting, Friedman analyses for ranks and post-hoc (Bonferroni) analyses were performed [28].

Changes in liking were analysed by comparing initial liking and wanting with the liking and wanting scores after the 3 week exposure. Paired sample t-tests were performed to investigate significant differences.

In order to investigate the association between food choice and liking, and food choice and wanting, two separate Anova's per snack food were carried out. Anova 1: independent variable = choice behaviour, dependent variables = liking before and liking after the intervention. Anova 2: independent variable = choice behaviour, dependent variables = wanting before and wanting after the intervention. Due to colinearity of liking and wanting, and the small number of subjects per group, measures of liking and wanting were not taken together in one Anova model (SPSS version 14). Choice behaviour in this matter was defined as: the number of times a particular snack was chosen during the 3-week intervention, by children in the CHOICE-group. P-values of less than 0.05 were considered statistically significant.

## Results

### Initial preference, liking and wanting

Before the intervention the products were differently preferred (F(4df) = 87.41; p < 0.0001) and liked (F(4df) = 65.10; p < 0.0001). Post-hoc analyses suggested that the Large chocolate-hazelnut snack and the Control snack were significantly less liked than the remaining snacks (all above 4 on a 5-point liking scale) (p < 0.05). Furthermore, the Large chocolate-hazelnut and the Control snacks were significantly less wanted than the remaining products (all above 3.5) (F(4df) = 101.35; p < 0.0001; post-hoc analyses p < 0.05) (see Table 2)

**Table 2:** Initial liking and wanting scores of all children for Small chocolate-hazelnut, Large chocolate-hazelnut, Small Sweet and Small Sweet-sour snacks, n = 242

Snack	Mean liking (± sem)	Mean wanting (± sem)
Small chocolate-hazelnut	4.04 ± 0.68	3.76 ± 0.8
Large chocolate-hazelnut	3.27 ± 0.09	3.07 ± 0.09
Small Sweet	4.06 ± 0.06	3.80 ± 0.07
Small Sweet-Sour	4.0 ± 0.06	3.81 ± 0.08
Control	3.60 ± 0.07	3.07 ± 0.09

### Consumption of products

On average children consumed between 89% and 97% of all the Small snacks (i.e. Small chocolate-hazelnut, Small Sweet and Small Sweet-Sour) they were offered during the three week intervention. Children who were asked to consume the Large chocolate-hazelnut snack for 3 weeks, ate between 67% and 85% of their daily servings, this was a statistically significant difference (F(3df) = 3.9; p < 0.05).

### Difference in liking before and after exposure

**Figure 2:**
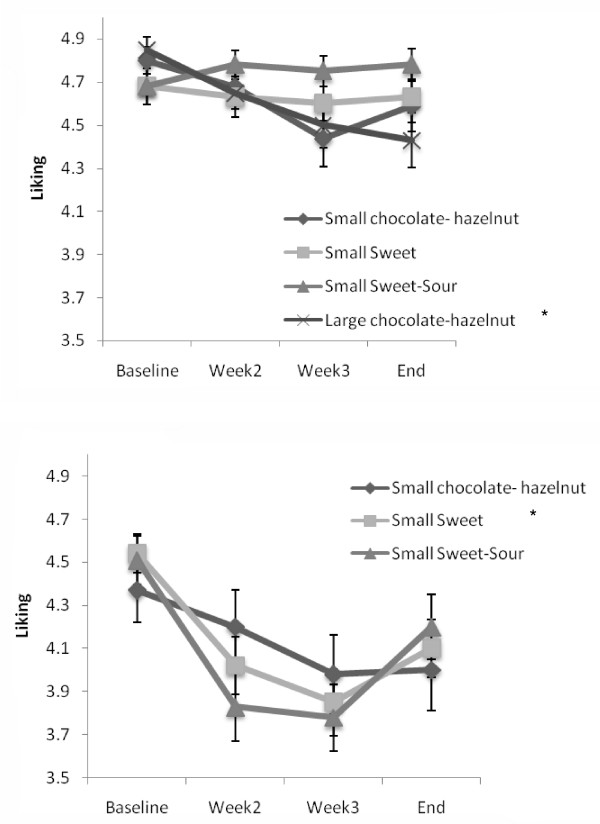
**Mean (± sem)change in liking (from 1 = not liked at all, to 5 extremely liked) during a daily consumption of the Small chocolate-hazelnut, Small Sweet, Small Sweet-Sour or Large chocolate hazelnut snack**. Shown for children who did not have a choice (upper panel) and children who could freely choose between the Small snacks (lower panel). * signifies significant decrease in liking from baseline to end p < 0.05.

As shown in Figure 2 upper panel differences in flavour (Sweet vs Sweet-Sour) did not result in a different change in liking. Children who consumed the Small Sweet as well as children who consumed the Small Sweet-Sour both reported a stable liking for the snacks they consumed throughout the intervention.

Differences where, however, observed depending on size of the snack food. That is, children who consumed the Large chocolate-hazelnut snack on a daily basis for three weeks, significantly decreased their liking for this snack (t(39df) = 3.19; p < 0.01). In contrast with children who consumed the Small chocolate-hazelnut snack for three weeks. They did not decrease their liking for this product during the intervention (t(40df) = 2.49; p = 0.10).

Children in the CHOICE-group tended to report a decrease in liking for all products they could choose from during the exposure period. This, however, only reached significance for the Small chocolate-hazelnut snacks (t(40df) = 2.88; p < 0.01) (Figure 2, lower panel). Children who were not exposed to any experimental product (control group) did not change their liking for any of the snack products. Furthermore the Control snack which was only offered at baseline and the end of the intervention did not change in liking or wanting.

### Difference in wanting before and after exposure

**Figure 3:**
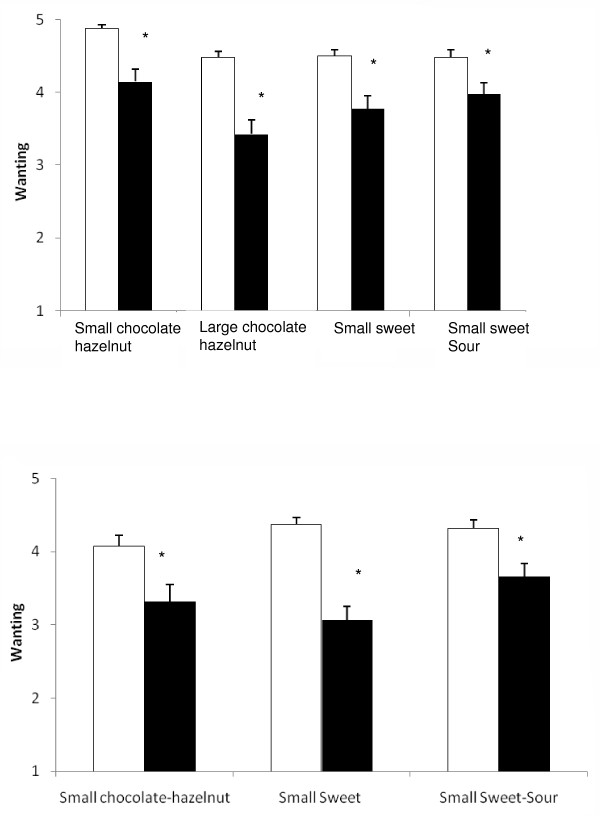
**Mean (± sem) Wanting (before (clear bars) and after (solid bars) a daily consumption of either the Small chocolate-hazelnut (n = 41), Large chocolate-hazelnut (n = 41), Small Sweet (n = 40) and SmallSweet-Sour (n = 40). **Shown for children who did not have a choice (upper panel) and children who could freely choose between the Small snacks (n = 40, lower panel).* signifies significant differences at P < 0.05

In contrast to liking, after children ate specific snacks daily for three weeks their wanting to eat these products decreased. This was independent from flavour, size (Figure 3, upper panel) or choice (Figure 3, lower panel) (all p-value's < 0.05). Children in the control group did not change their wanting for any of the products.

### CHOICE group: Free choice during three weeks

**Figure 4:**
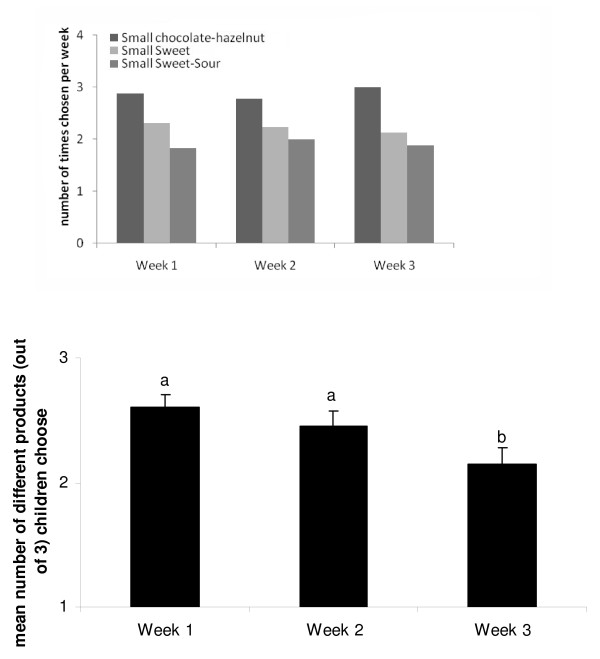
**Upper panel- Mean number of times (± sem) children in the CHOICE group choose either Small chocolate-hazelnut, Small Sweet or Small Sweet-Sour snacks during the first, second and third week of the intervention (min = 0, max = 7)**. Lower panel- Mean number of different products (± sem) (out of 3: Small, Small Sweet, Small Sweet-Sour) children in the free CHOICE group (n = 37) choose during week 1, 2 and 3.

Data from 4 subjects in the CHOICE group were incomplete, because parents failed to fill out which products were chosen each day. This resulted in 37 complete records of children in the CHOICE group. Only a few children (5.4%, n = 2) did not switch between products during the 3 weeks of the intervention and always choose the Small chocolate-hazelnut snack to consume. Most children (32.4%) switched between the three Small snack foods which were made available to them. As shown in Figure 4 upper panel, on average the Small chocolate-hazelnut snack was chosen the most often across the three weeks of intervention (on average 2.9 times out of 7). On average children's variety of snacks consumed was higher in the first week compared to the last week (F(3df) = 7.66; p < 0.05) (Figure 4 lower panel). Younger children (7 to 9 years of age) compared to older aged children (10 to 12 years of age), choose a large variety during the first week (t = 2.1, p < 0.05). No differences were observer for the second week (t = 0.95, p = 0.35), third week (t = -0.43, p = 0.67) or total variety across three week (t = 0.98, p = 0.42).

### Liking and wanting as predictors for choice

As shown in Table 3, the liking after the 3 week intervention (with the liking-before-the intervention as covariate) rather than the liking before the intervention was a significant correlate of food choice for all three snack foods. The wanting after 3 week intervention (with the wanting-before-the intervention as covariate) showed to be a correlate for food choice for two out of the three snacks (Small chocolate hazelnut and Small sweet sour). When comparing the statistically significant effect sizes (B) of liking and wanting on food choice, it can be concluded that the effect size of liking on food choice is consistently larger than the effect size of wanting on food choice

**Table 3:** Anova 1-upper panel: association food choice (dependent variable) and liking (independent variables).

	model		Liking			
	
			before			after
						
	F	p	B	95%CI	B	95%CI
	
Small chocolate hazelnut	21.0	<0.001	0.65	-1.4 – 2.8	3.8	2.5–5.0
Small sweet	3.3	<0.05	0.58	-1.8 – 3.0	2.0	0.33–3.6
Small sweet-sour	9.0	<0.01	0.18	-1.4 – 1.7	2.5	1.3 – 3.7
						

	model		Wanting			
	
			before			after
						
	F	p	B	95%CI	B	95%CI
	
Small chocolate hazelnut	14.0	<0.001	1.4	-0.26 – 3.1	2.5	1.4–3.5
Small sweet	0.49	0.62	0.68	-2.0 – 3.3	0.58	-0.70 – 1.9
Small sweet-sour	8.2	<0.001	0.38	-1.3 – 2.0	2.1	1.1 – 3.2

Anova 2- lower panel: association food choice (dependent variable) and wanting (independent variables). Data for children in the CHOICE group (n = 37). Food choice is defined as the number of times children in the CHOICE group choose a particular snack during the 3 week intervention.

## Discussion

This study investigated the influence of repeated consumption of snack foods on children's liking and wanting. This was tested with products which differed in Sweet-Sour balance, or size in two different conditions (monotonous and free choice condition). First, it will be discussed how repeated consumption of snacks influenced changes in liking and wanting in general. Subsequently, it will be discussed how size (i.e. Small chocolate-hazelnut vs Large chocolate-hazelnut), flavour profile (Sweet vs Sweet-Sour), and freedom of choice influenced changes in liking and wanting.

Previous studies showed that children increased their liking over repeated consumption of novel foods such as different cheeses [29] or novel vegetables [30]. In these studies initial liking for these novel foods was moderate to low, possibly due to children's food neophobic responses [31]. It has been shown that liking for novel foods increases after repeated consumption, because the novel foods become more familiar to children [32]. This contrasts findings of the present study as liking of the snack products remained relatively stable over time. Since these products were very recently introduced to the Turkish market, we may assume that children had no or little exposure to these foods prior to their participation in our study. Lack of increase in liking after repeated consumption might be due to a ceiling effect because most products were highly liked at the start of the intervention.

The present study hypothesised that small sized snacks resulted in less decrease of liking but not wanting, over daily consumption for three weeks, than large sized snacks. In the present study repeated consumption of small sized snack foods indeed seemed to show a less decrease in liking than a repeated consumption of large sized snack foods. Wanting, however, showed a similar decrease for small and large sized snack foods. At the same time children consumed more of the Small chocolate-hazelnut snacks during the three week intervention than of the Large chocolate-hazelnut snacks.

The latter finding is in contrast to Weijzen et al's study which focussed on sensory specific satiety in adults [22]. They suggested that adults consumed 12% less of small sized food than large sized foods when given the snacks during a one meal time occasion [22]. The present study and Weijzen's study are, however, different. It has been suggested that children, compared to adults show different chewing behaviour. That is, adults have a higher masticatory performance and a higher bite force than children [33]. Therefore, it may cost children more effort than adults to eat large snack foods compared with small snack foods. This may explain why children eat more of the small snack foods than of the large snack foods over the course of three weeks. Furthermore, Weijzen's study concerned sensory specific satiety which can be seen as an intra meal measurement of boredom. In the present study boredom and intake was measured over a course of three weeks. To date it is unclear, to our knowledge, whether decreases in liking during one meal occasions are related to decreases in liking after a prolonged exposure (i.e 3 weeks).

The effect of size on a decrease in liking might be related to the amount of oral stimulation. Children may eat small sized snacks faster than large sized snacks because they need less mastication and a lower bite force to breakdown the food before swallowing. It can be argued that when eating at a high rate, the food stays in the mouth during a shorter time than when eating slowly. Fast eating gives the sensory receptors in the mouth less time to interact with flavour and texture. This generates less sensory satiety of the sensory receptors [22,34]. This is still highly speculative because we did not measure speed of eating in the current study.

The present study hypothesised that after daily consumption for three weeks, the liking and wanting of sweet snack foods would increase and the liking and wanting of sour snack foods would remain stable. In the present study, Sweet-Sour balance did not influence long term liking, wanting or choice. A previous study with beverages which differed in sweet-sour balance found that repeated exposure to the sweet beverages increased liking for this beverage over time. Whereas repeated exposure to the sour beverages did not result in a different pattern of liking [10]. It could be that children were not able to taste differences between the sweet and sweet sour snack. However, a small pilot with adults showed that 6 out of 8 people reported the Sweet-Sour snack to be more sour than the Sweet snack. Children, however, might have been less sensitive to these small differences in taste than adults. An alternative explanation could be that the Sweet and the Sweet-Sour snacks were highly liked. In order for sweet-sour balance to be able to increase long term liking, the foods may need to be moderately liked as was the case in the previous study [10].

In adults it has generally been found that liking *decreases *after repeated exposure for a variety of foods (see [35] for review). In the present study most products, except for the Large snacks, remained stable in liking. This suggests that on average children's liking remains stable for at least three weeks. Potentially 3 weeks was not enough to show a decrease in liking. Le et al [21], however, did also not observe a change in liking for noodle soup when children were exposed to this soup for 10 weeks. A decrease in liking for highly liked food might not be evident in children. This may prevent children from trying out foods they never tried, which could impact their dietary variety [36].

Wanting, however, decreased. This was most likely due to the repeated exposure. Recall that children who did not receive a repeated exposure to a particular snack food, did not change their wanting for these foods. The decrease in wanting was specific for flavour and size. For example, children who daily consumed the small snack with chocolate hazelnut flavour decreased their wanting for this particular snack but not for those snack with either a similar size or flavour. This suggests that in order to prevent a decrease in wanting, flavours and sizes may need to be rotated during the week. This does not mean that children should be given a wide variety of choice each day.

In the present study it was hypothesised that children who could freely choose between snack products which differed in flavour and size would express a lower decrease in liking and wanting, over daily consumption for three weeks, than those who were not given a choice. In contrast to our expectations, free choice did not prevent decrease in liking and wanting in children. Recall that children who were allowed to freely choose between 3 types of Small snacks tended to decrease their liking for these snacks during the course of three weeks. In adults it has generally be found that giving consumers a choice between different flavours prevented boredom with the products [12]. This is most likely due to consumers' feeling of control [23]. Possibly, children felt pressured by the given choice (i.e. you have to choose) which negatively impacted upon their liking and wanting. As pointed out by Schwartz, making a choice may make us realize that we missed out on the options we did not choose, which result in a lower satisfaction of the one we did choose [37]. In western societies children are overwhelmed by choice. Crisps, soda and many other products come in multiple flavours. Our research suggests that a large choice may have a negative effect on liking and wanting of any one specific product.

When children were given a choice we observed that after trying different flavours during the first week of exposure, children seem to pick their favourite and remain eating this snack for the remainder of the intervention. This suggests that initial success of different foods developed for children, might be misleading. Children may not continue eating a high variety of different foods but rather narrow down their choices. This might also be true for much younger children than we tested. Nicklaus and colleagues found that children decrease the number of different foods they eat between 2 and 3 years of age [38].

In the present study initial liking or wanting was not the strongest predictor of food choice. Food choice was best explained by the change in liking across the three weeks intervention. Furthermore, children who initially preferred the Large snacks tended to show a stronger decline in liking and wanting during the three week intervention than those who initially preferred the Small snacks. This suggests that consumer tests with children which select potential successful products based on initial liking may fail to select products which will be successful in the market in the long term. This may also be the case for adults [38].

Neither initial wanting nor the change in wanting played a significant role in food choice. This does not mean that wanting is irrelevant for children's food related behaviours. Recently it has been suggested that obese children showed a higher wanting for foods than lean peers [39]. Differences in children's liking for particular tastes are rarely observed [36]. Similar results have been obtained in adults [40]. It has previously been suggested that wanting depends on contextual factors such as the context in which a particular food is given (or not given), and the perceived appropriateness of consumption of particular foods [14]. Wanting can therefore fluctuate depending on the context in which a food is provided. Liking, as shown in the present study and previous studies [38], is more stable. It can be hypothesised that liking determine the range of food which are acceptable, whereas wanting plays a dominant role in which food will be eaten and in which quantity. This, however, needs to be tested in future research.

The present study had several limitations. In order to try to mimic a real life situation children were given the snacks they initially liked most. Regression to the mean effect could have accounted for differences observed between the first measurement of liking and subsequent measurements. After children were grouped based on their liking for the different snacks it seems that those in the Large-snack food group scored the large snack foods as extremely liked. Because of this, they are more likely to decrease their liking for this food than when the food had not been extremely liked, as was the case for the remaining groups. However, it needs to be noted that the present data suggest that the liking of the large snack foods gradually decreased over the course of three weeks. Children in the choice group were not selected based on their initial liking but rather comprised of a random sample of all children. Regression to the mean would therefore have little effect on changes in liking for this group. But it is this group which showed a decrease in liking for all products they were exposed to.

Consumption was not individually measured. Therefore we could not asses the relationship between liking & wanting and food consumption. Future studies should aim to investigate this relationship by measuring intake per individual rather than on a group level.

During the intervention period snacks were consumed at their children's home in their natural environment. By doing this we tried to minimize the impact of the researchers and lab-environment on children's rating of liking and wanting. Therefore we had no control about how the products were consumed (e.g whether children's played with their food, how long it took to eat). Parents were, however given strict instructions about how and when to offer the snacks to the children.

Furthermore, in order to investigate the intrinsic properties of the products (smell, taste, texture), products were provided unbranded. In real life, extrinsic product properties such as brand and nutritional messages are likely to have a large influence on children food choice [41-43]. Future studies should therefore focus on extrinsic properties as well.

## Conclusion

The present study suggests that children's liking for large sized snack foods is more likely to decrease after a daily consumption than identically flavoured Small sized snacks. It needs to be investigated whether same principles hold true for foods which are not highly liked such as vegetables. We hypotheses that smaller sized foods encourage children to eat these food repeatedly due to the lower amount of effort involved than when eating large sized foods.

Decrease in liking during daily consumption of the same food was a better predictor of food choice than initial liking. Therefore a liking test with children based on a once off tasting may not represent market success. Wanting decreased more after daily consumption than liking. It remains to be determined how this decrease in wanting affects children's food consumption. Sensory testing with children should therefore not only focus on liking, but rather on liking & wanting.

Furthermore, choice appears to have a negative effect on liking. It is therefore recommended to offer children a limited choice rather than an unlimited choice.

## Competing interests

The authors declare that they have no competing interests.

## Authors' contributions

DGL designed the study, analyzed the data, and conceived and drafted the original manuscript. EHZ provided critical feedback on study design and drafts of the manuscript. All authors read and approved the final manuscript.
